# Supplementary material for: Virtual brain twins for stimulation in epilepsy
Source: Nat Comput Sci. 2025 Aug 5;5(9):754–68. doi: 10.1038/s43588-025-00841-6 (PMC12457187; doi:10.1038/s43588-025-00841-6)
Supplement: Supplementary file 1 — Supplementary Figs. 1–10 and Descriptions of Videos 1–9. [file 43588_2025_841_MOESM1_ESM.pdf]

nature computational science

Supplementary information

https://doi.org/10.1038/s43588-025-00841-6

Virtual brain twins for stimulation in epilepsy

In the format provided by the authors and unedited

Contents

1 Supplementary information: Virtual brain twins 1

2 Quantification of SEEG and TI Stimulation Effects 2

3 Supplementary Figures 3

4 Supplementary movies 13

1 Supplementary information: Virtual brain twins

We developed a virtual brain twins pipeline tailored for stimulation based on personalized whole brain network modeling in epilepsy. This pipeline integrates empirical patient data and undergoes validation using simulated data generated from high-resolution network models. They are able to explore stimulation effects. Moreover, the proposed pipeline employs model inversion through HMC and Bayesian inference to estimates EZNs. We demonstrated the estimation of the EZN from SEEG and scalp-EEG signals during seizure activity triggered by direct electrical SEEG or TI stimulation. We introduced multimodal inference integrating multiple, potentially simultaneous, modalities as well. The virtual brain twins pipeline for stimulation provides a directly applicable methodological and conceptual basis for a series of scientific studies and clinical translations. The concept of virtual brain twins from this pipeline can be extended to other brain disorders, such as Alzheimer's and psychiatric disorder, among others [1].

The virtual brain twins for stimulation incorporate three levels of personalization, adding one more level compared to previous versions [1, 2]. These include: (1) individual geometry and connectome derived from anatomical data such as MRI, (2) estimation of key parameters, such as spatial distribution of excitability (related to EZN), from functional data such as SEEG and EEG, (3) personalized stimulation protocol. The electric field calculations are based on individual MRI data, taking into account brain geometry and head tissue segmentation. The personalized spatial distribution of excitability provides a tailored response to the stimulation protocol, enhancing the effectiveness of the intervention.

The informative prior plays an important role in the calculations of the posterior, enabling reliable and efficient evaluation of potential hypotheses [3]. In the EPINOV clinical trial, we used the informative prior derived from the data analysis of SEEG onset features [2] in maximum a posterior (MAP) estimation algorithm. We also tested

whether the brain sodium MRI-derived prior could slightly improve the performance of VEP [4]. In this paper, we further improved our model inversion algorithm, demonstrating that even with an uninformative prior, the algorithm can still identify the targeted EZN. The effectiveness is due to the current model inversion algorithm, which includes reparameterization and a combination of both MAP estimation and the HMC algorithm.

2 Quantification of SEEG and TI Stimulation Effects

We quantitatively compared the effects of SEEG and TI stimulation based on source-level brain activity through the high-resolution brain activity simulations. These effects were characterized by spatial distribution and activation amplitude. Spatial distribution was assessed using the activated area and the largest activated distance. The stimulation effects immediately after stimulation stops are shown in Supplementary Fig.8 . SEEG stimulation leads to a larger seizing area (Supplementary Fig. 8**a,c**) but a similar maximal distance among these areas (Supplementary Fig. 8**b**). In contrast, TI stimulation induced a lower activation amplitude (Supplementary 8**d**).

One immediate application is to help design and verify that non-invasive stimulation achieves similar focality and effects as invasive stimulation. For example, the optimization of multipolar TI stimulations [5] promises to be as effective as invasive deep brain stimulation for Parkinson's disease and epilepsy. Efficiency can be defined in terms of spatial distribution (focality) and stimulated amplitude.

3 Supplementary Figures

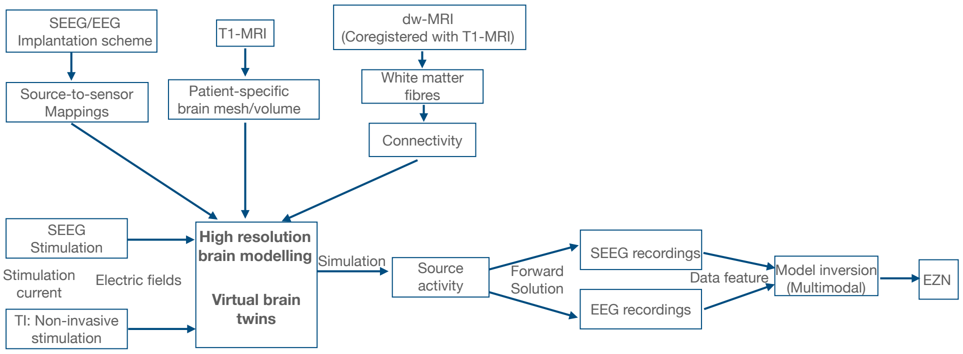

Supplementary Fig. 1 |Flowchart of the virtual-brain-twin stimulation pipeline. First, a T1-MRI defines the patient-specific high-resolution space. Second, the structural connectivity, the gain matrix (source-to-sensor matrix), and sensor-to-source matrix are obtained in a patient-specific brain space by co-registering both diffusion-weighted MRI and CT for SEEG locations and standard EEG system configuration with T1-MRI. We used two types of stimulation: SEEG and Temporal interference (TI). The electric field generated by the stimulation current influences the brain activity which can be generated by the high-resolution personalized whole-brain model. We extract data features from SEEG recordings and EEG recordings from both SEEG-stimulation and TI stimulation and use HMC techniques for model inversion. From each stimulation module we can obtain a posterior distribution of epileptogenic values, which suggests EZN candidates. Then the integration module combines multiple results in different cases to help the clinical decision.

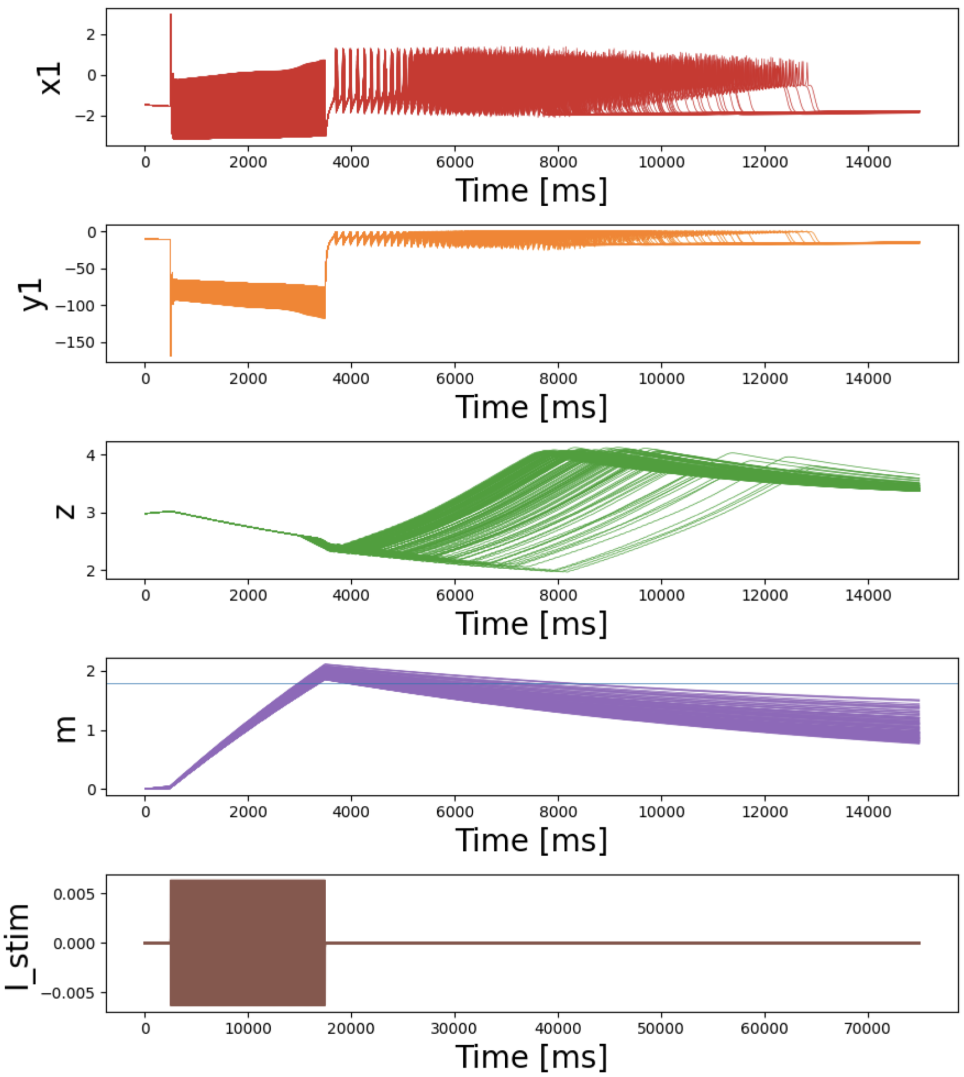

Supplementary Fig. 2 |The time-series of Epileptor stimulation variables during induced seizures through SEEG stimulation. The five state variables of the model at the seizing parts of the cortex, along with the stimulation time course I_{stim}. The stimulation I_{stim} were rescaled based on the electric field, allowing seizure induction. The stimulation is accumulating on state variable m of the Epileptor-Stimulation equation (1). Once m passes a fixed threshold it causes the model to initiate the seizure.

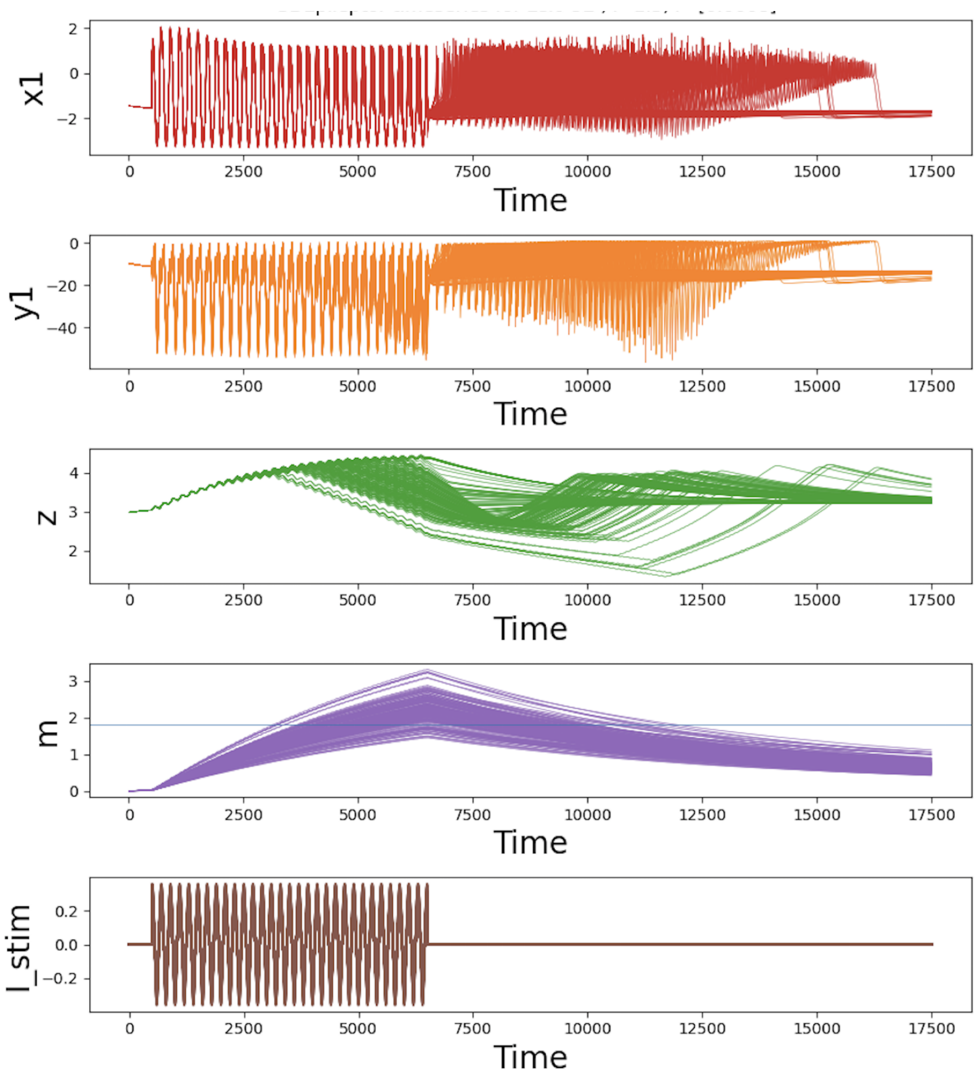

Supplementary Fig. 3 |The time-series of Epileptor-Stimulation equations by inducing seizure through stimulation induced by TI. The five state variables of the model at the seizing parts of the cortex, along with the stimulation time course I_{stim}. The stimulation I_{stim} were rescaled based on the electric field, allowing seizure induction. The stimulation is accumulating on state variable m of the Epileptor. Once m passes a fixed threshold it causes the model to initiate the seizure.

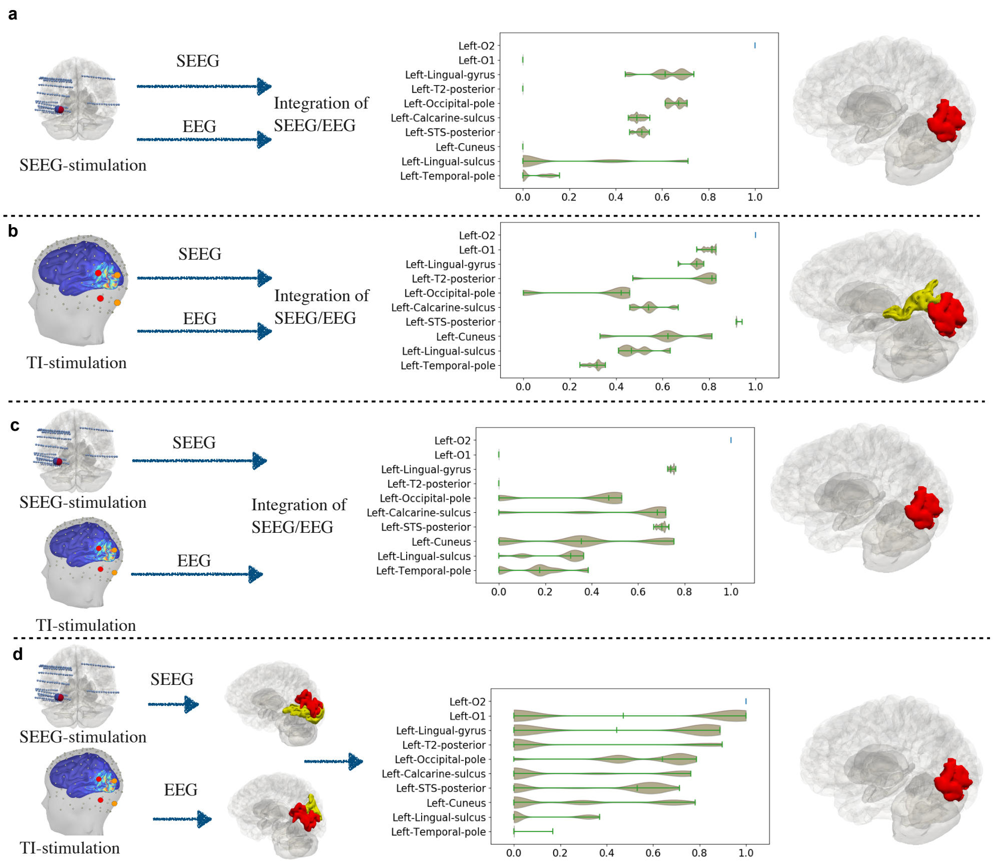

Supplementary Fig. 4 |Integration module for multiple SEEG and scalp-EEG recordings.(**a-c**) The posterior distribution of EVs from using the HMC on a simultaneous SEEG and scalp-EEG data, which were mapped from the same high resolution source data. This model inversion can be performed in three cases: seizures induced by SEEG stimulation (**a**, the same case in Fig. 5**a**), by TI (**b**, the same case in Fig. 5**b**) and by both SEEG and TI (**c**). (**d**) A combination of the posterior distribution of EVs by pooling the EVs distribution obtained from SEEG (Fig. 3**d** under SEEG stimulation) and scalp-EEG recordings (Fig. 4**d** under TI stimulation). Each row shows the data used for model inversion, the posterior of the EVs and the corresponding highlighted EZs on the 3D brain, where the left O2 is in red and left STS-posterior is in yellow.

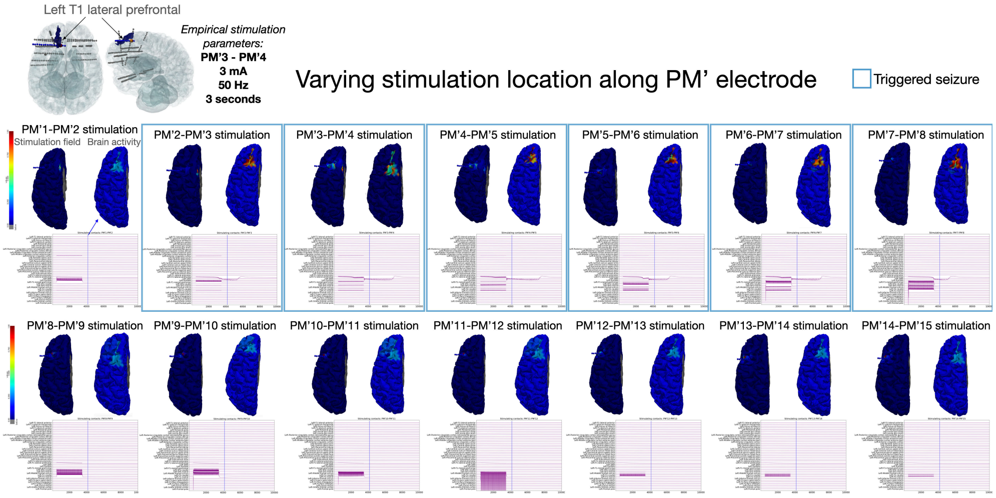

Supplementary Fig. 5 |Stimulation location was varied alongside the PM' electrode, while all other stimulation parameters remained constant. Fourteen cases are represented. Top Left: the stimulation field for each of the corresponding stimulation locations. Top Right: the resulting brain activity shortly after stimulation, measured at the time point marked in blue in the time series (bottom). The stimulation effects, applied from $t = 500$ ms to $t = 3500$ ms, are visible in all cases but vary depending on the stimulation site. In six out of fourteen electrode pairs, a seizure was triggered in the epileptogenic region 'Left-T1-lateral-prefrontal'. In these cases, the stimulating electrodes were sufficiently close to this area to trigger a seizure. In all other cases, the stimulating electrodes were too far from EZNs to trigger a seizure in the regions where they generated a focal electric field, due to those regions having a higher seizure threshold than the EZNs.

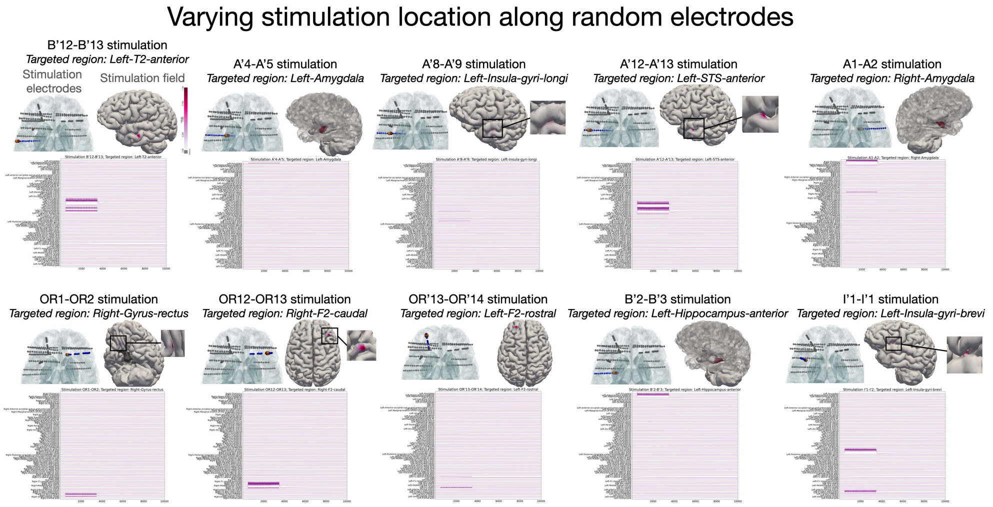

Supplementary Fig. 6 |Stimulation location varied across different electrodes, while all other stimulation parameters remained constant. Ten cases are represented. In each case: Top Left—stimulation electrode highlighted in blue and stimulating pair shown in red and orange. Top Right—estimated stimulation field from the corresponding stimulation location. Bottom-simulated time series of brain activity before, during and after stimulation. The stimulation effects, applied from $t = 500$ ms to $t = 3500$ ms, are visible in all cases but vary depending on the stimulation site. Different non-epileptogenic brain regions were targeted by the focal stimulation field and no seizure was triggered.

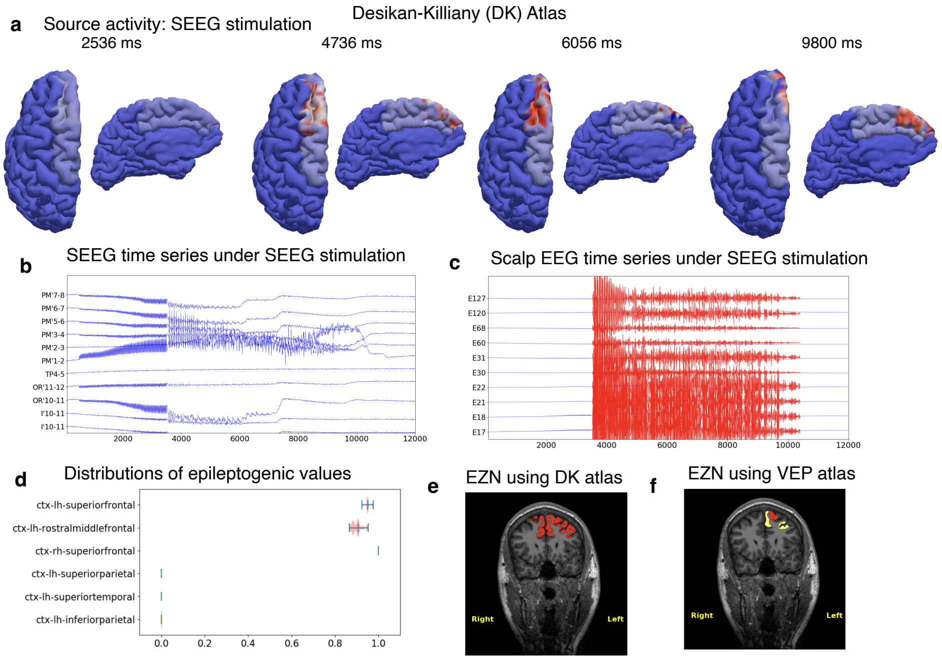

Supplementary Fig. 7 |Simulation and estimating EZN in the pipeline using the Desikan-Killiany (DK) atlas. (**a**) Snapshot of source activity (corresponding videos available in the supplementary materials) at four specified time instants under SEEG stimulation using DK atlas. (**b**) Selected simulated SEEG time-series and (**c**) Scalp EEG time-series from SEEG stimulation-induced seizure. The scaled-up time series during the seizure period is shown in red. **d** Posterior distribution of EVs from HMC sampling when analyzing simultaneously SEEG and scalp-EEG using DK atlas. (**e**) Heatmap of the estimated EZN in T1-MRI from the pipeline using the DK atlas, based on results from (**d**). (**f**) Heatmap of estimated EZN in T1-MRI from the pipeline using the VEP atlas, based on results from Fig. 6 (**d**).

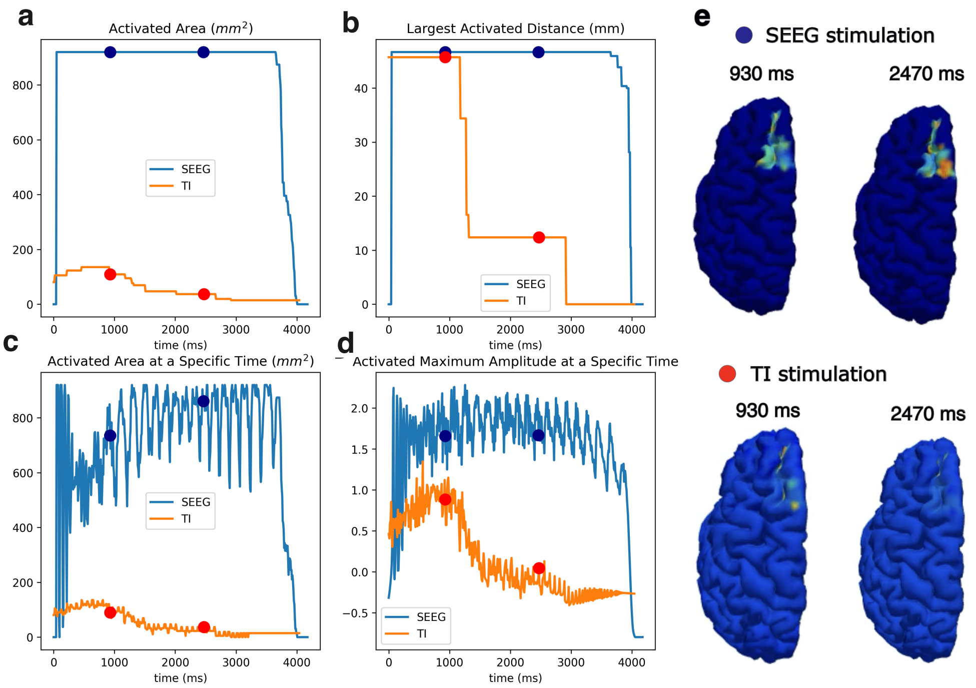

Supplementary Fig. 8 |Comparison of the activated stimulation effect immediately after stimulation stops between SEEG and TI stimulation. $t = 0$ represents the moment when stimulation stops. Blue indicates SEEG stimulation, while orange represents TI stimulation." **(a)**. We first identified the seizure onset and offset times for vertices that show seizure activity at the source level. Then, we calculated the activated area at a specific time by identifying vertices where this specific time falls between their seizure onset and offset. **(b)**. The largest activated distance at a specific time is defined as the maximum distance between all activated vertices where this specific time falls between their seizure onset and offset. **(c)**. Unlike **a**, here the activated vertices are defined based on whether they are active at a specific time point. **(d)**. The mean amplitude of the top 10 vertices, ranked by their amplitudes. **(e)**. The brain activity at the source level simulation under SEEG and TI stimulation at two example time points, 930 ms and 2470 ms, marked as dots in **a-d**. The clips are extracted from the same videos as Fig. 6 **b** and Fig. 7 **b**.

.

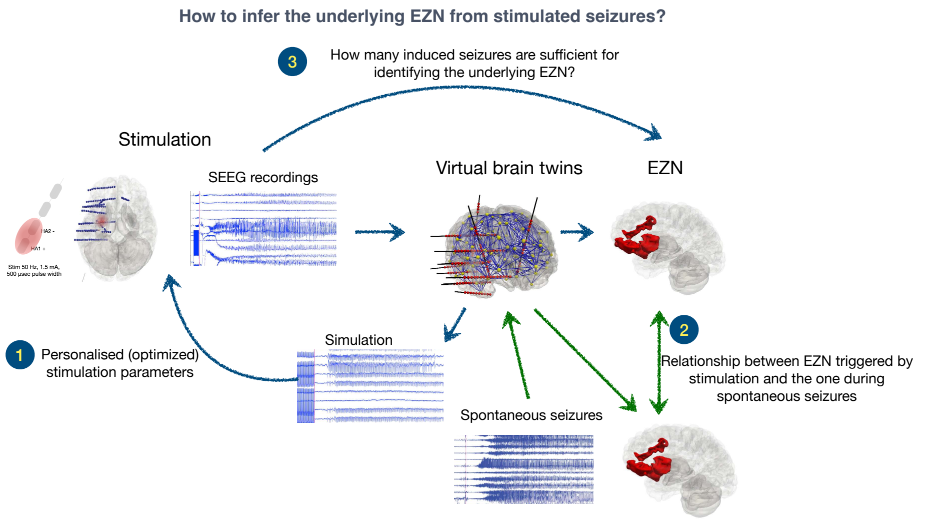

Supplementary Fig. 9 |The study scheme to answer: How to infer the underlying EZNs from stimulated seizures? We illustrated three related systematic studies and details described in the first paragraph of subsection of future scientific studies.

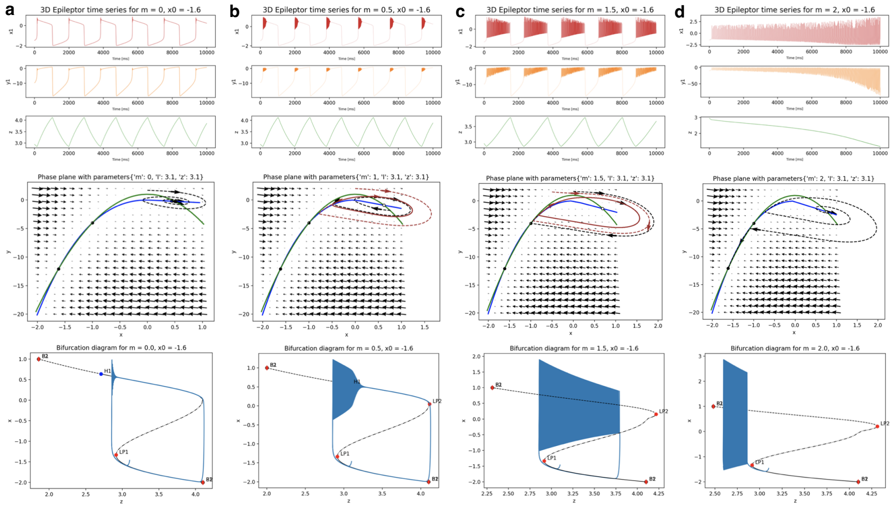

Supplementary Fig. 10 |Varying m in a single Epileptor model. The 3D Epileptor model with varying m. Each row, from top to bottom: time series for each variable (x_1, y_1, z), phase plane plot with nullclines x_1 and y_1 for $z = 3.1$ and a complete bifurcation diagram for varying values of z (dotted line: unstable fixed points, continuous line: stable fixed points). (**a**) $m = 0$, the oscillations in the upstate are brief because the equilibrium in the upstate is a stable spiral. (**b**) $m = 0.5$, oscillations in the upstate are of higher amplitude and longer because the equilibrium in the upstate is an unstable spiral with a limit cycle (in red in the phase plane plot). (**c**) $m = 1.5$, oscillations in the upstate have an even higher amplitude and last for the entire duration of the upstate, the limit cycle, shown in red in the phase plane has a greater diameter. (**d**) $m = 2$, the system exhibits unstable spiral behaviour.

4 Supplementary movies

Movie 1: The brain activity of Patient 1 under SEEG stimulation on high-resolution brain surfaces. The bipolar SEEG stimulation initiates at 505 ms, lasting for 3 seconds. A seizure is induced around 3,505 ms and terminates around at 10,255 ms. Observing seizure activity at the source level can help our understanding epileptogenic zones.

Movie 2: The brain activity of Patient 1 under temporal interference stimulation on high-resolution brain surfaces. The temporal interference stimulation initiates at 500 ms, lasting for 6 seconds. A seizure is induced at approximately 3,505 ms and terminates around 13,400 ms.. Observing seizure activity at the source level can help our understanding epileptogenic zones.

Movie 3: The brain activity of Patient 2 under SEEG stimulation on high-resolution brain surfaces. The bipolar SEEG stimulation initiates at 504 ms, lasting for 3 seconds. A seizure is induced around 3,504 ms and concludes at 10,072 ms. Observing seizure activity at the source level can help our understanding epileptogenic zones.

Movie 4: The brain activity of Patient 2 under temporal interference stimulation on high-resolution brain surfaces. The temporal interference stimulation initiates at 1,000 ms, lasting for 2 seconds. A seizure is induced around 3,000 ms and concludes at 7,660 ms. Observing seizure activity at the source level can help our understanding epileptogenic zones.

Movie 5-8: The brain activity of Patient 2 under SEEG stimulation on high-resolution brain surfaces, with local connectivity within the EZN is r times the local connectivity of other brain regions with r=2,3,4,5.

Movie 9: The brain activity of Patient 2 under SEEG stimulation on high-resolution brain surfaces, using the Desikan-Killiany atlas.

References

[1] Wang, H. E. *et al.* Virtual brain twins: from basic neuroscience to clinical use. *National Science Review* **11** (2024).

[2] Wang, H. E. *et al.* Delineating epileptogenic networks using brain imaging data and personalized modeling in drug-resistant epilepsy. *Science Translational Medicine* **15** (2023).

[3] Hashemi, M. *et al.* On the influence of prior information evaluated by fully bayesian criteria in a personalized whole-brain model of epilepsy spread. *PLOS Computational Biology* **17**, e1009129 (2021). URL https://dx.plos.org/10.1371/journal.pcbi.1009129.

[4] Azilinon, M. *et al.* Brain sodium mri-derived priors support the estimation of epileptogenic zones using personalized model-based methods in epilepsy. *Network Neuroscience* 1–41 (2024).

[5] Zhu, X. *et al.* Multi-point temporal interference stimulation by using each electrode to carry different frequency currents. *IEEE Access* **7**, 168839–168848 (2019).
