# Supplementary material for: Virtual brain twins for stimulation in epilepsy
Source: Nat Comput Sci. 2025 Aug 5;5(9):754–68. doi: 10.1038/s43588-025-00841-6 (PMC12457187; doi:10.1038/s43588-025-00841-6)
Supplement: Supplementary file 2 — Reporting Summary [file 43588_2025_841_MOESM2_ESM.pdf]

nature portfolio

Corresponding author(s): Huifang Wang and Viktor Jirsa

Last updated by author(s): May 10, 2025

Reporting Summary

Nature Portfolio wishes to improve the reproducibility of the work that we publish. This form provides structure for consistency and transparency in reporting. For further information on Nature Portfolio policies, see our Editorial Policies and the Editorial Policy Checklist.

Statistics

For all statistical analyses, confirm that the following items are present in the figure legend, table legend, main text, or Methods section.

n/a	Confirmed	
☐	☒	The exact sample size (*n*) for each experimental group/condition, given as a discrete number and unit of measurement
☐	☒	A statement on whether measurements were taken from distinct samples or whether the same sample was measured repeatedly
☒	☐	The statistical test(s) used AND whether they are one- or two-sided<br>*Only common tests should be described solely by name; describe more complex techniques in the Methods section.*
☒	☐	A description of all covariates tested
☐	☒	A description of any assumptions or corrections, such as tests of normality and adjustment for multiple comparisons
☐	☒	A full description of the statistical parameters including central tendency (e.g. means) or other basic estimates (e.g. regression coefficient) AND variation (e.g. standard deviation) or associated estimates of uncertainty (e.g. confidence intervals)
☒	☐	For null hypothesis testing, the test statistic (e.g. *F*, *t*, *r*) with confidence intervals, effect sizes, degrees of freedom and *P* value noted<br>*Give P values as exact values whenever suitable.*
☐	☒	For Bayesian analysis, information on the choice of priors and Markov chain Monte Carlo settings
☐	☒	For hierarchical and complex designs, identification of the appropriate level for tests and full reporting of outcomes
☒	☐	Estimates of effect sizes (e.g. Cohen's *d*, Pearson's *r*), indicating how they were calculated

Our web collection on statistics for biologists contains articles on many of the points above.

Software and code

Policy information about availability of computer code

Data collection	Siemens Magnetom Verio 3T MR-scanner for T1-weighted imaging and diffusion MRI images, Deltamed/Natus system for SEEG recordings.
Data analysis	FreeSurfer 6. 3.0: Used for volumetric segmentation and cortical surface reconstruction; https://surfer.nmr.mgh.harvard.edu/fswiki/DownloadAndInstall<br>Cortical surface parcellation: https://github.com/HuifangWang/VEP_atlas_shared.git<br>Mrtrix 0.3.16 software package for processing DW-MRI data. https://mrtrix.readthedocs.io/en/0.3.16/<br>GARDEL 1.0 (Graphical user interface for Automatic Registration and Depth Electrodes Localization) for location of the SEEG contacts from post-implantation CT scans. https://meg.univ-amu.fr/doku.php?id=epitools:gardel<br>SIMNIBS 4.0: Used for electric field calculation.; https://simnibs.github.io/simnibs/build/html/index.html<br>Brainstorm3:Forward solution for scalp-EEG signals; https://neuroimage.usc.edu/brainstorm/Installation#Requirements

For manuscripts utilizing custom algorithms or software that are central to the research but not yet described in published literature, software must be made available to editors and reviewers. We strongly encourage code deposition in a community repository (e.g. GitHub). See the Nature Portfolio guidelines for submitting code & software for further information.

Data

Policy information about availability of data

All manuscripts must include a data availability statement. This statement should provide the following information, where applicable:

- Accession codes, unique identifiers, or web links for publicly available datasets
- A description of any restrictions on data availability
- For clinical datasets or third party data, please ensure that the statement adheres to our policy

This work involves two types of data: raw clinical data, including T1-MRI and DWI-MRI images, CT scans, and SEEG data. The raw data are available upon request, but they are not central to this paper. All derived data and key information for personalized modeling will be publicly available after publication.

Human research participants

Policy information about studies involving human research participants and Sex and Gender in Research.

Reporting on sex and gender	We studied two patients:<br>1) A 23-year-old female diagnosed with left occipital lobe epilepsy. She underwent resective surgery and was nearly seizure-free post-surgery, with an Engel Class II outcome.<br>2) A 19-year-old male diagnosed with left frontal lobe epilepsy. He underwent resective surgery resulting in complete seizure freedom, with an Engel Class I outcome.
Population characteristics	We have two patients: a 23-year-old female with occipital lobe epilepsy and a 19-year-old male with frontal lobe epilepsy. One had a surgical outcome classified as Engel Class I, and the other as Engel Class II.
Recruitment	We used two epilepsy patients with drug resistant focal epilepsy who underwent a standard presurgical protocol at La Timone hospital in Marseille. We selected these two patients to validate our workflow. Future scientific studies should include a broader range of epilepsy types and surgical outcomes.
Ethics oversight	Informed written consent was obtained for all patients in compliance with the ethical requirements of the Declaration of Helsinki and the study protocol was approved by the local Ethics Committee<br>(Comité de Protection des Personnes sud Méditerranée 1)

Note that full information on the approval of the study protocol must also be provided in the manuscript.

Field-specific reporting

Please select the one below that is the best fit for your research. If you are not sure, read the appropriate sections before making your selection.

☒ Life sciences ☐ Behavioural & social sciences ☐ Ecological, evolutionary & environmental sciences

For a reference copy of the document with all sections, see nature.com/documents/nr-reporting-summary-flat.pdf

Life sciences study design

All studies must disclose on these points even when the disclosure is negative.

Sample size	This is a methodology and concept paper. We selected two patients with drug-resistant focal epilepsy. These two patients have different diagnoses and surgical outcomes. For each patient, the dataset includes both anatomical and functional data from multiple recordings, such as T1-weighted MRI, CT, diffusion-weighted MRI, and multiple stereo-EEG sessions. Since this is a methodology and proof-of-concept paper for personalized medicine, we believe that an in-depth analysis of two patients is sufficient to demonstrate the feasibility of our approach.
Data exclusions	No data were excluded from the analyses
Replication	The results are replicable if the same parameters were used on the same datasets.The results are replicable when the same parameters are applied to the same datasets. Since this study focuses on personalized virtual brain twins, replication is expected during the construction of virtual twins or the identification of the epileptogenic zone networks in similar cases.
Randomization	Because this study focuses on personalized medicine using patient-specific data, randomization is not necessary.
Blinding	The VEP analysis is independent of the patients' clinical hypothesis and surgery outcomes. Blinding was not applicable in this context for several reasons. First, the study is retrospective and all clinical interventions had already been completed prior to analysis. Second, the Virtual Epileptic Patient (VEP) analysis is computational and data-driven, conducted independently of clinical outcomes or hypotheses. Finally, since the methodology relies on objective modeling of individualized brain dynamics rather than subjective interpretation, the lack of blinding does not compromise the validity or integrity of the results.

Reporting for specific materials, systems and methods

We require information from authors about some types of materials, experimental systems and methods used in many studies. Here, indicate whether each material, system or method listed is relevant to your study. If you are not sure if a list item applies to your research, read the appropriate section before selecting a response.

Materials & experimental systems

n/a	Involved in the study
☒	☐ Antibodies
☒	☐ Eukaryotic cell lines
☒	☐ Palaeontology and archaeology
☒	☐ Animals and other organisms
☐	☒ Clinical data
☒	☐ Dual use research of concern

Methods

n/a	Involved in the study
☒	☐ ChIP-seq
☒	☐ Flow cytometry
☐	☒ MRI-based neuroimaging

Clinical data

Policy information about clinical studies

All manuscripts should comply with the ICMJE guidelines for publication of clinical research and a completed CONSORT checklist must be included with all submissions.

Clinical trial registration: It is not a clinical trial. The clinical data is being used for a research study.

Study protocol: The goal of the study is to develop and evaluate the VEP high-resolution workflow for stimulation. VEP is a personalized workflow, so we selected two patients with drug-resistant focal epilepsy, each with different diagnoses and surgical outcomes. Using these patients' data, we built personalized whole-brain models. These models can predict the effects of stimulation and can be further used to better estimate epileptic networks.

Data collection: For each patient, the dataset includes both anatomical and functional data from multiple recordings, such as T1-weighted MRI, CT, diffusion-weighted MRI, and multiple stereo-EEG sessions.

Outcomes: The VEP models can predict the effects of stimulation and can be further used to better estimate epileptic networks.

Magnetic resonance imaging

Experimental design

Design type: No functional MRI data was recorded, only structural and diffusion weighted images.

Design specifications: Not used.

Behavioral performance measures: Not used.

Acquisition

Imaging type(s): presurgical T1 weighted MRI, presurgical diffusion weighted MRI, post SEEG implantation CT scan

Field strength: 3 Tesla

Sequence & imaging parameters: MPRAGE sequence, repetition time = 1.9 or 2.3 s, echo time = 2.19 or 2.98 ms, voxel size 1.0 mm3, FoV full head
CT scans FoV full head, voxel size around 0.4mm * 0.4mm * 0.6mm

Area of acquisition: Whole brain scan

Diffusion MRI: ☒ Used ☐ Not used

Parameters: Either single shell, b-values = [0,1000], 64 directions or multi-shell, b-values = [0, 1400, 1800], 200 directions, no cardiac gating used

Preprocessing

Preprocessing software: Freesurfer v6, FSL v6, MRtrix 0.3.16

Normalization: No spatial normalization was used in this study as all processing, modeling and inference is done in the imaging space of each individual patient.
Only a linear registration was performed to align between patient specific T1, diffusion and CT images.

Normalization template	Not used.
Noise and artifact removal	T1 weighted was processed using the recon-all pipeline from Freesurfer.<br>Diffusion weighted MRI was processed using the functionality of the MRtrix software package.
Volume censoring	No volume censoring performed.

Statistical modeling & inference

Model type and settings	Not used.
Effect(s) tested	Not used.

Specify type of analysis: ☒ Whole brain ☐ ROI-based ☐ Both

Statistic type for inference<br>(See Eklund et al. 2016)	Not used.
Correction	Not used.

Models & analysis

n/a	Involved in the study
☒	☐ Functional and/or effective connectivity
☒	☐ Graph analysis
☒	☐ Multivariate modeling or predictive analysis
